# Control of the polarization direction of isolated attosecond pulses using inhomogeneous two-color fields

**DOI:** 10.1038/s41598-019-54984-4

**Published:** 2019-12-09

**Authors:** Stephen Maina Njoroge, Hua Yuan, Kinyua Dickson, Qingbin Zhang, Pengfei Lan

**Affiliations:** Wuhan National Laboratory for Optoelectronics and School of Physics, Huazhong University of Science and Technology, Wuhan, 430074 China

**Keywords:** High-harmonic generation, Attosecond science

## Abstract

We theoretically demonstrate the control of the polarization direction of isolated attosecond pulses (IAPs) with inhomogeneous two-color fields synthesized by an 800-nm fundamental pulse and a 2000-nm control pulse having crossed linear polarizations. The results show that by using the temporally and spatially shaped field, the high-order harmonic generation (HHG) process can be efficiently controlled. An ultra-broad supercontinuum ranging from 150th to 400th harmonics which covers the water window region is generated. Such a supercontinuum supports the generation of a 64-as linearly polarized IAP, whose polarization direction is at about 45° with respect to the x axis. Moreover, we analyze the influence of the inhomogeneity parameters and the relative angle of the fundamental and control pulses on the IAP generation. It is shown that the polarization direction of the IAP can rotate in a wide range approximately from 8° to 90° relative to the x axis when the inhomogeneity parameters and the relative angle vary.

## Introduction

High-order harmonic generation (HHG) is a nonlinear interaction of an intense laser field with matter. In the past years, HHG has been widely investigated both in theory and experiment since it offers an effective way to produce extreme ultraviolet (XUV) and soft x-ray sources as well as generate attosecond pulses^1,2^. The attosecond pulses are important tools to study and control the ultrafast electron dynamics in atoms^3–16^, molecules^17–24^, nanostructures^25–28^ and solids^29–36^ with an extremely high temporal resolution. This has stimulated both HHG and the generation of attosecond pulses. Many methods have been proposed to produce isolated attosecond pulses (IAPs), such as few-cycle laser pulses, the polarization gating technique, and two-color or multicolor fields^37–40^.

HHG in noble gases requires the laser intensity of the order of 10^13^W/cm^2^ ^41^. Such intensity is beyond the output power of a convectional femtosecond oscillator, which is in the range of 10^11^–10^12^ W/cm^2^ ^25,26^. To exceed the intensity threshold for HHG, the chirped pulse amplification (CPA) system is usually adopted. However, the amplification requires several regenerative and multi-pass amplifier cavities^41,42^. Recently, the plasmonic field-enhanced HHG in the vicinity of metallic nanostructures has attracted much attention^25,26,43–50^. The output beam from a femtosecond oscillator is directly focused onto a nanostructure. The intensity of the incident field could be enhanced by more than two orders without extra cavities due to the surface plasmon resonances. The enhanced field is sufficient for the HHG process to occur. Moreover, in the nanogap where HHG takes place, the enhanced field is spatially inhomogeneous. HHG driven by such an inhomogeneous field shows some novel characteristics^43–50^, such as the generation of even order harmonics and the selection of the quantum path. In particular, by using the inhomogeneous field, the harmonic spectrum can be effectively extended, even to the water window region^50,51^, which has an important application for imaging living specimens in biology. Recently, the generation of IAPs in the inhomogeneous field has been proposed^49,50^.

As for the IAP, the polarization characteristics, such as the polarization state and the polarization direction, are of great importance for its practical applications. The previous studies were mainly devoted to the generation of linearly polarized (LP) attosecond pulses. Recently, circularly (or largely elliptically) polarized attosecond pulses have found important uses in chiral recognition, magnetic circular dichroism to time-resolved magnetization dynamics and spin currents^52–55^. This has stimulated much effort to produce the circularly polarized (CP) attosecond pulses, for examples, bichromatic circularly polarized fields, two CP counter-rotating pulses in a non-collinear geometry^56,57^. Nevertheless, the control of the polarization direction of the IAPs has been seldomly investigated. In principle, the control of the polarization direction of the attosecond pulse can be easily accomplished by changing the polarization direction of a LP single-color field. However, this scheme may be difficult to generate a broadband supercontinuum as well as an ultra-short IAP^46,50^.

In this paper, we theoretically demonstrate control of the polarization direction of IAPs with the cross-LP inhomogeneous two-color field, which is synthesized by an 800-nm fundamental pulse and a 2000-nm control pulse. Based on the quantum results obtained by solving the two-dimensional time-dependent Schrödinger Eq. (2D-TDSE), we show that the HHG can be efficiently controlled by using the inhomogeneous two-color field. Then a 250-th (150–400th) supercontinuum covering the water window region and a 64-as IAP with polarization direction at about 45° with respect to the x axis are produced. Moreover, we have also discussed the influence of the inhomogeneity parameters and the relative angle of the two pulses on the polarization direction of the generated IAPs. The results show that the polarization direction of IAPs can vary in a wide range approximately from 8° to 90° relative to the x axis_._

## Theoretical Model

In our simulations, the harmonic spectra are obtained by solving 2D-TDSE, which is given by (atomic units are used throughout this paper)

1 $$\begin{array}{rcl}i\frac{\partial \psi (x,y,t)}{\partial t} & = & H(x,y,t)\psi (x,y,t)\\  & = & [-\frac{1}{2}\frac{{\partial }^{2}}{\partial {x}^{2}}-\frac{1}{2}\frac{{\partial }^{2}}{\partial {y}^{2}}+{V}_{a}(x,y)+{V}_{l}(x,y,t)]\psi (x,y,t).\end{array}$$

Here ${V}_{a}(x,y)=-\,\frac{1}{\sqrt{{x}^{2}+{y}^{2}+\alpha }}$ is the soft-core potential. The soft core parameter α is chosen to be 0.1195 to match the ground ionization potential of neon atom which is 0.7925 a.u (21.6 eV). ${V}_{l}(x,y,t)=-\,{E}_{x}(x,t)x-{E}_{y}(y,t)y$ is the potential due to laser-electron interaction. The x, y components of the inhomogeneous field are given by

2 $$\begin{array}{rcl}{E}_{x}(x,t) & = & {E}_{xt}(t)(1+{\varepsilon }_{x}x),\\ {E}_{y}(y,t) & = & {E}_{yt}(t)(1+{\varepsilon }_{y}y).\end{array}$$

Here the parameters *ε*_*x*_ and *ε*_*y*_ define the strengths of the spatial inhomogeneity of the laser field along x, y directions, respectively^44,48,50^.

In our calculations, an 800-nm fundamental pulse and a 2000-nm control pulse with crossed linear polarizations are adopted to synthesize the two-color field. The intensities of these two laser pulses are chosen to be $3.0\times {10}^{14}\,W/c{m}^{2}$ and $6.0\times {10}^{13}\,W/c{m}^{2}$, respectively. The electric fields of the x, y components of the two-color fields can be written as

3 $$\begin{array}{rcl}{E}_{xt}(t) & = & {E}_{0}f(t)\cos ({\omega }_{0}t+{{\varphi }}_{0})+{E}_{1}f(t)\cos ({\omega }_{1}t+{{\varphi }}_{1}-\varDelta {\varphi })\cos \,\theta ,\\ {E}_{yt}(t) & = & {E}_{1}f(t)\cos ({\omega }_{1}t+{{\varphi }}_{1}-\varDelta {\varphi })\sin \,\theta .\end{array}$$

Here *E*_0_, *E*_1_, *ω*_0_, *ω*_1_ *ϕ*_0_ and *ϕ*_1_ are the amplitudes, frequencies and carrier-envelope phases (CEPs) of the fundamental and control pulses, respectively. θ and Δ*ϕ* are the relative angle and the relative phase between the two pulses, respectively. $f(t)={\sin }^{2}(\pi t/T)$ is the laser envelope. *T* = 10*T*_0_, *T*_0_ is the optical cycle of the fundamental pulse.

We use the split-operator method to solve Eq. (1) ^58^. To avoid spurious reflections from the boundaries, the electron wavefunction is multiplied by a mask function at each time step^59^. The neon atom is in the initial (ground) state before the laser is turned on. The ground state is obtained by imaginary time propagation with the soft-core potential. Once the electron wavefunction $\psi (x,y,t)$ is obtained, the time-dependent dipole acceleration along x and y direction is calculated by the Ehrenfest theorem^60^

4 $$\begin{array}{rcl}{a}_{x}(t) & = & -\langle \psi (x,y,t)|[H(x,y,t),[H(x,y,t),x]]|\psi (x,y,t)\rangle ,\\ {a}_{y}(t) & = & -\langle \psi (x,y,t)|[H(x,y,t),[H(x,y,t),y]]|\psi (x,y,t)\rangle .\end{array}$$

The HHG spectrum is obtained by Fourier transforming time-dependent dipole acceleration,

5 $$\begin{array}{rcl}{|{s}_{qx}(\omega )|}^{2} & = & {|\frac{1}{T}{\int }_{0}^{T}{a}_{x}(t){e}^{-iq\omega t}dt|}^{2},\\ {|{s}_{qy}(\omega )|}^{2} & = & {|\frac{1}{T}{\int }_{0}^{T}{a}_{y}(t){e}^{-iq\omega t}dt|}^{2}.\end{array}$$

The attosecond pulse can be obtained by superposing several orders of harmonics,

6 $$\begin{array}{rcl}{I}_{x}(t) & = & {|\sum _{q}{s}_{qx}{e}^{iq\omega t}dt|}^{2},\\ {I}_{y}(t) & = & {|\sum _{q}{s}_{qy}{e}^{iq\omega t}dt|}^{2}.\end{array}$$

Here q is the harmonic order.

## Results and Discussion

**Figure 1:**
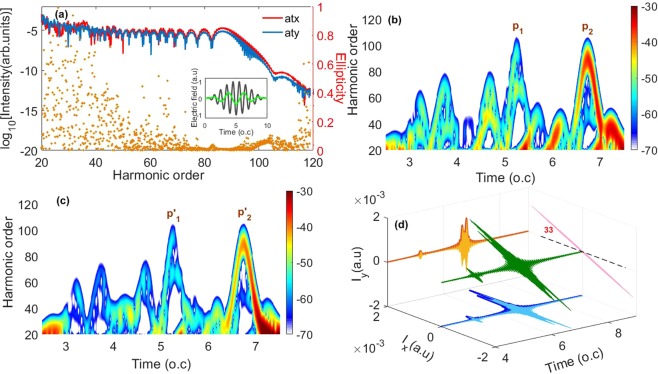
(**a**) The x, y components (the red and blue lines) of the generated harmonic spectrum in the homogeneous two-color field. The ellipticity of harmonics is also presented as the yellow dot. The inset shows the electric fields of the fundamental (the black line) and control (the green line) pulses. (**b**,**c**) Time-frequency distributions of the x, y components of the harmonic spectrum, respectively. Here, the time-frequency distribution is plotted by the linear scale. The numbers on the right of the color scale is the amplitude of the time-dependent dipole acceleration. (**d**) The 3D plot of the electric field of the IAP generated by superposing 75th to 90th harmonics.

Figure 1(a–c) show the x, y components of the calculated harmonic spectrum and corresponding time-frequency distributions in the homogeneous (*ε*_*x*_ = *ε*_*y*_ = 0) two-color laser field. The ellipticity of harmonics is also presented (the yellow dot) in Fig. 1(a). Here, the fundamental and control pulses are cross-polarized with the relative angle θ of 0.3π. The relative phase and CEPs of these two laser pulses are set to be 0. The electric fields of the fundamental (the black line) and control (the green line) pulses are shown in the inset of Fig. 1(a). As can be seen from Fig. 1(b), the harmonics above 70th are contributed by two emission peaks, *P*_1_ and *P*_2_. Among them, the peak *P*_2_ has much higher intensity than *P*_1_, it dominates the harmonic emission, therefore the harmonics above 70th become continuous. The maximum energy of *P*_2_ is at the 90th harmonic, corresponding to the harmonic cutoff. Besides, the peak *P*_2_ has two branches: the short and long quantum paths. The interference of these quantum paths leads to the modulation structure of the x component of the supercontinuum (70th–90th) (the red line), as shown in Fig. 1(a). Similar results have also been found in the y component of the harmonic spectrum. Due to the interference of the short and long quantum paths of the peak *P*′_2_ which has much higher intensity than *P*′_1_ [see Fig. 1(c)], the y component of the supercontinuum above 70th presents modulation structure [see the blue line in Fig. 1(a)]. Moreover, as shown in Fig. 1(a), the harmonic ellipticity of the supercontinuum is 0 (the yellow dot). By superposing the harmonics from 75th to 90th around in the supercontinuum, we can get a LP attosecond double pulse due to the presence of the short and long quantum paths. The polarization direction of the generated LP attosecond double pulse is approximately 33° relative to the x axis [see Fig. 1(d)].

**Figure 2:**
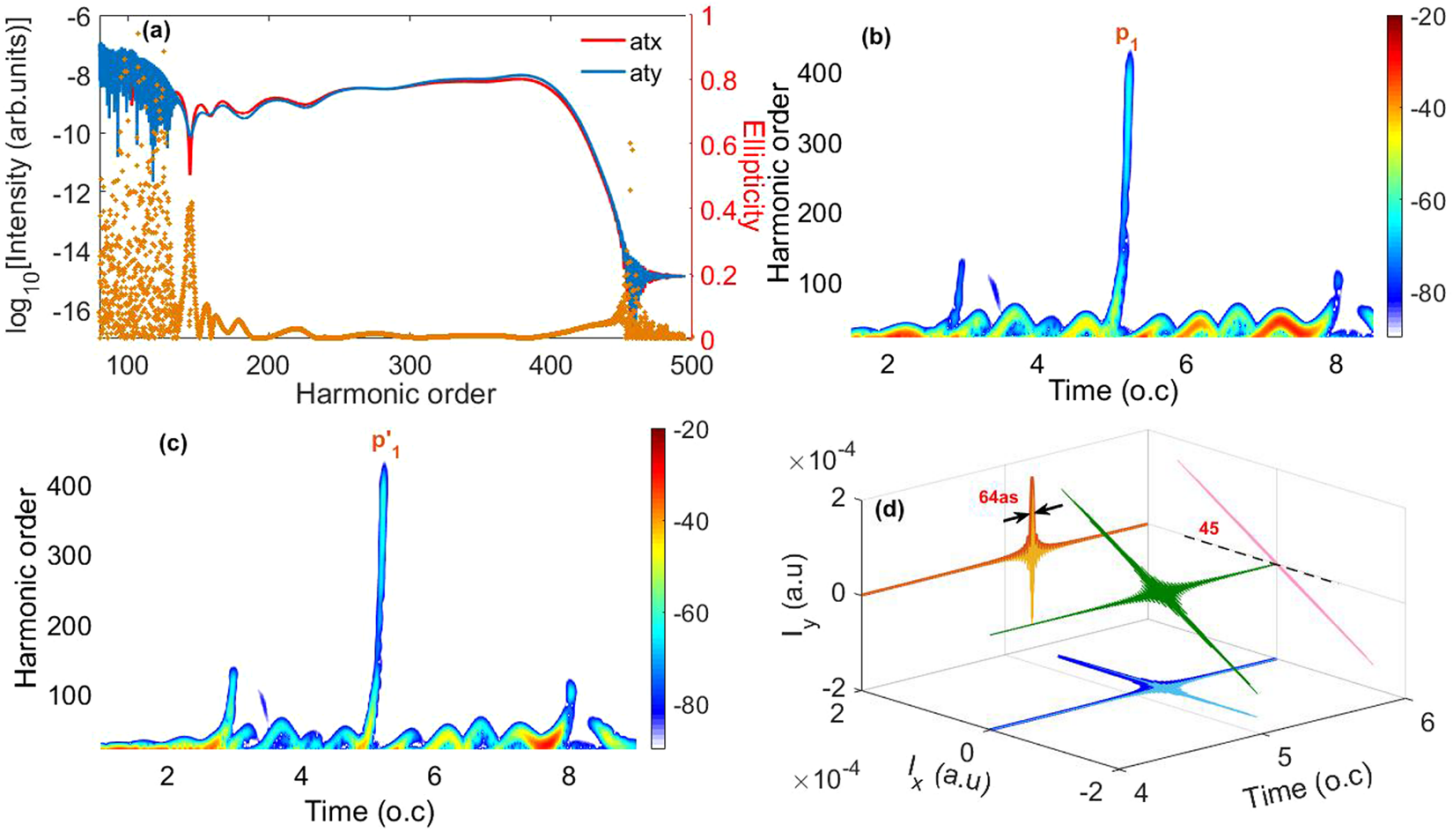
(**a**) The x, y components (the red and blue lines) of the generated harmonic spectrum in the inhomogeneous two-color field with the inhomogeneity parameters *ε*_*x*_ = 0.003 and *ε*_*y*_ = 0.01, respectively. The harmonic ellipticity is also presented as the yellow dot. (**b**,**c**) Time-frequency distributions of the x, y components of the harmonic spectrum, respectively. Here, the time-frequency distribution is plotted by the linear scale. The numbers on the right of the color scale is the amplitude of the time-dependent dipole acceleration. (**d**) The 3D plot of the electric field of the IAP generated by superposing 250th to 300th harmonics. Except for the inhomogeneity parameters, other parameters are the same as those in Fig. 1(a).

To generate an IAP, we consider modulating the electron dynamics by using a cross-LP inhomogeneous two-color field. Here, the inhomogeneity parameters of the fundamental and control pulses are taken as *ε*_*x*_ = 0.003 and *ε*_*y*_ = 0.01, respectively. Other parameters are the same as in Fig. 1(a). Figure 2(a) shows the x, y components of the generated harmonic spectrum in the two-color inhomogeneous field. One can see that the intensities of the x, y components of the harmonic spectrum are comparable. But the harmonic cutoffs are extended to the 400th harmonic and a smooth supercontinuum with a bandwidth of 250th harmonics [from 150th to 400th] covering the water window region is successfully produced. For a deeper insight, we present the corresponding time-frequency distributions in Fig. 2(b–c). One can see that the harmonics above 150th are contributed by only one emission peak *P*_1_ and *P*′_1_ for the x and y harmonic components, respectively. The maximum energy of the highest emission peaks *P*_1_ and *P*′_1_ reaches 400th harmonic, corresponding to the harmonic cutoff. Moreover, only the short quantum paths survive for the peaks *P*_1_ and *P*′_1_. Therefore a broadband 250th (from 150th to 400th) supercontinuum which spans the water window region, is generated [see Fig. 2(a)]. Furthermore, the harmonic ellipticity of the supercontinuum is still 0 (the yellow dot). By superposing harmonics from 250th to 300th in the supercontinuum, a 64-as LP IAP is obtained, as shown in Fig. 2(d). The polarization direction of the LP IAP is at about 45° relative to x axis [see Fig. 2(d)]. Note that we choose the harmonics in a window of 50 harmonic orders in width to synthesize the attosecond pulse throughout the paper in order to guarantee that an IAP can be generated in the inhomogeneous field when the inhomogeneity parameters and relative angle vary.

**Figure 3:**
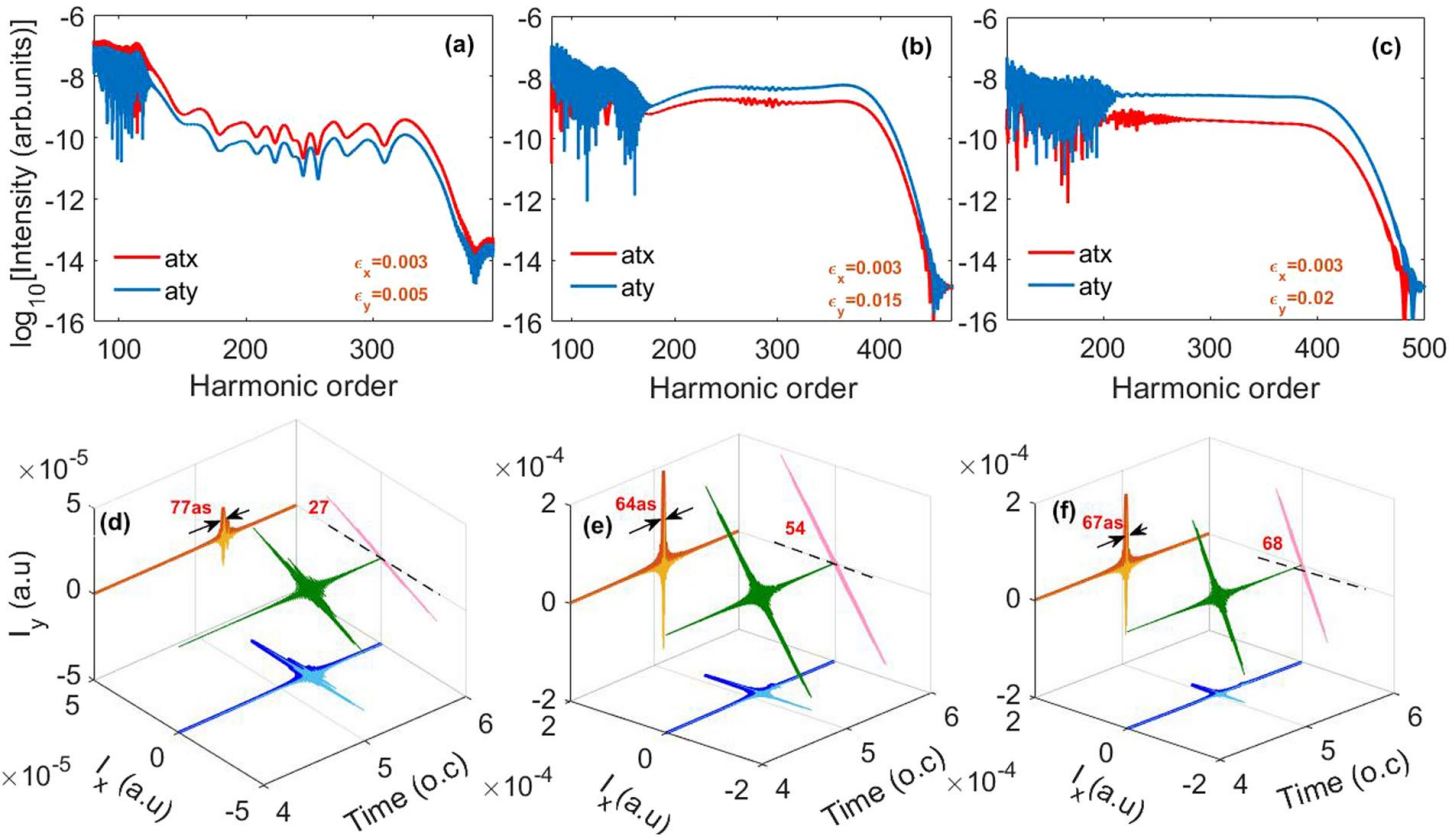
(**a**,**d**) The x, y components of the generated harmonic spectrum and the 3D plot of the electric field of the IAP generated by superposing 250th to 300th harmonics in the inhomogeneous two-color field with the inhomogeneity parameter along y direction *ε*_*y*_ of 0.005. (**b**,**e**,**c**,**f**) are same as (**a**,**d**), but for the cases of *ε*_*y*_ = 0.015 and *ε*_*y*_ = 0.02, respectively. Except for *ε*_*y*_, other parameters are the same as those in Fig. 2(a).

In Fig. 3(a–c), we discuss the influence of the inhomogeneity along y direction on the generated harmonic spectra in the two-color inhomogeneous field. The generated IAPs by superposing 250th to 300th harmonics are also presented in Fig. 3(d–f). Here, the inhomogeneity parameter *ε*_*y*_ of the y component of the electric field is chosen to be 0.005, 0.015 and 0.02, respectively. Other parameters are the same as those in Fig. 2(a). One can see that the cutoff extension and smooth supercontinuum generation are robust against the variation of the inhomogeneity. However, the intensities of the x, y components of the generated harmonic spectrum are no longer the same in comparison with the case of *ε*_*y*_ = 0.01 [shown in Fig. 2(a)]. For the case of *ε*_*y*_ = 0.005 shown in Fig. 3(a), the intensity of the x harmonic component is higher than that of the y harmonic component. By superposing harmonics from 250th to 300th, an IAP with the duration of 77as, whose polarization direction is about 27° relative to the x axis, is generated [as shown in Fig. 3(d)]. While in the case of *ε*_*y*_ = 0.015, the y harmonic component has higher intensity than the x harmonic component [as shown in Fig. 3(b)]. An IAP with the duration of 64as and the polarization direction of about 54° relative to the x axis is generated [see Fig. 3(e)]. As *ε*_*y*_ increases to 0.02, the magnitude difference between the x, y components of the generated harmonic spectrum is further enlarged. Therefore, the polarization direction of the generated IAP also changes, which is at about 68° relative to the x axis. The duration of the generated IAP is 67as. It is worth noting that the generation of the broadband supercontinuum and ultra-short IAP with different inhomogeneity parameters in the cross-LP inhomogeneous two-color field is similar to that in the commonly used parallel-polarized inhomogeneous two-color field^49,50^. However, the polarization direction of the IAP can change with the variation of the inhomogeneity parameters in the cross-polarized configuration while the variation of inhomogeneity parameters may not change the polarization direction of the IAP in the parallel-polarized configuration.

**Figure 4:**
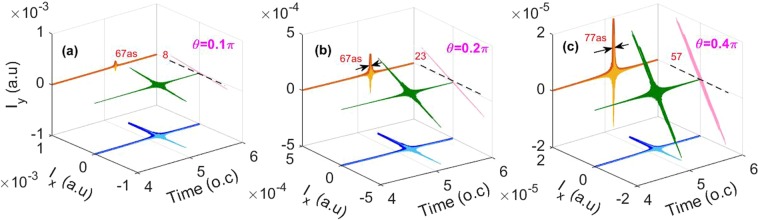
The 3D plot of the electric field of the IAP generated by superposing 250th to 300th harmonics for the cases of the relative angle θ between the fundamental and control pulses of (**a**) 0.1π, (**b**) 0.2π and (**c**) 0.4π, respectively. Except for θ, other parameters are the same as those in Fig. 2(a).

In Fig. 4, we also investigate the effect of the relative angle θ between the fundamental and control pulses on the generation of the IAP. In our simulations, the relative angle θ is chosen to be 0.1π, 0.2π, and 0.4π, respectively. Other parameters are the same as those in Fig. 2(a). The IAPs are generated by superposing the harmonics from 250th to 300th in the water window region. One can see that LP IAPs with the duration below 80as are generated for all the relative angle values. Moreover, the polarization directions of the generated IAPs are at about 8°, 23° and 57° with respect to the x axis, when the relative angle θ is chosen to be 0.1π, 0.2π, and 0.4π, respectively.

**Figure 5:**
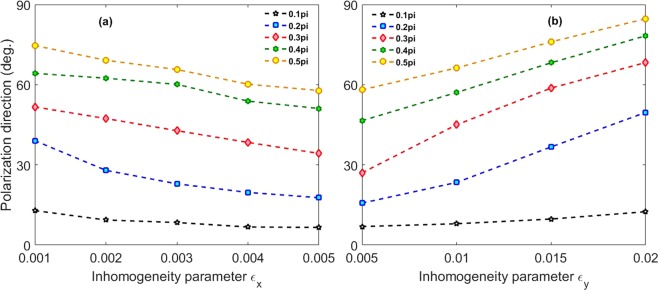
(**a**) Polarization direction of the generated IAP with different inhomogeneity parameter *ε*_*x*_ and relative angle θ. Here, *ε*_*y*_ is 0.01. (**b**) Polarization direction of the generated IAPs with different inhomogeneity parameter *ε*_*y*_ and relative angle θ. Here, *ε*_*x*_ of 0.003. Except for *ε*_*x*_, *ε*_*y*_, θ, other parameters are the same as those in Fig. 2(a).

Finally, in Fig. 5, we present variation of the polarization direction of the generated IAPs in the inhomogeneous two-color field when the inhomogeneity parameters *ε*_*x*_, *ε*_*y*_ and the polarization angle θ vary. In Fig. 5(a) the IAPs are generated by superposing harmonics from 200th to 250th harmonics while in Fig. 5(b) the IAPs are obtained by superposing harmonics from 250th to 300th harmonics. It is obvious that the polarization directions of the generated IAPs change with the variations of the inhomogeneity parameters and the polarization angle of the two-color fields, which can rotate in a wide range approximately from 8° to 90° with respect to the x axis.

## Conclusion

In conclusion, we have theoretically demonstrated the control of the polarization direction of the LP IAP using the two-color laser field having the crossing linear polarization. Based on the numerical solution of the 2D-TDSE, we show that HHG process can be efficiently controlled with the temporally and spatially shaped laser fields. An ultra-broad supercontinuum ranging from 150th to 400th which covers the water window region is obtained. By filtering out harmonics from 250th to 300th, a LP IAP in the water-window region with the duration of 64as is generated, whose polarization direction is at approximately 45° relative to the x axis. Moreover, the influence of the inhomogeneity parameters of the fundamental and control pulses and the relative angle between these two pulses on the generated IAP has also been investigated. It shows that the polarization directions of the generated IAPs can rotate in a wide range approximately from 8° to 90° with respect to the x axis when the inhomogeneity parameters and the relative angle vary.
